# Incorporation of L-Carnitine in the OvSynch protocol enhances the morphometrical and hemodynamic parameters of the ovarian structures and uterus in ewes under summer climatic conditions

**DOI:** 10.1186/s12917-023-03814-x

**Published:** 2023-11-24

**Authors:** Haney Samir, Ayman A. Swelum, Elshymaa A. Abdelnaby, Hossam R. El-Sherbiny

**Affiliations:** 1 Department of Theriogenology, Faculty of Veterinary Medicine, Cairo University, Giza, 12211 Egypt; 2 Department of Theriogenology, Faculty of Veterinary Medicine, Zagazig University, Zagazig, Egypt

**Keywords:** Sheep, OvSynch, Heat stress, L-Carnitine, Ovarian and uterine blood flow, Doppler.

## Abstract

Heat stress negatively impacts the reproductive performance of sheep including the efficiency of estrous synchronization regimens. This study aimed to investigate the potential effects of L-Carnitine (LC) administration on the efficacy of the OvSynch protocol in ewes under summer climatic conditions. Ewes were synchronized for estrus using the OvSynch protocol and a dose of LC (20 mg/kg body weight) was intravenously (IV) administered on the same day of PGF_2α_ injection to one group (n = 8; LC group), while other ewes (n = 8; control group) received the same protocol without LC. Ultrasonographic evaluation (including B-mode, color, and pulsed Doppler) was used to assess the morphometrical and hemodynamic parameters of ovarian structures [number, size, and blood flow of follicles (GFs) and corpora lutea (CLs)] and uterus during the estrous phase (Day 0), and on Day 8 post ovulation (luteal phase). Uterine artery blood flow (MUA) was assessed by measuring the resistive index (RI) and pulsatility index (PI) at both stages. The serum samples were collected to measure the concentrations of estradiol (E2), progesterone (P4), and total antioxidant capacity (TAC) using commercial kits. Results revealed a significant (P<0.05) increase in the colored pixel area of GFs and uterus in the LC group (392.84 ± 31.86 and 712.50 ± 46.88, respectively) compared to the control one (226.25 ± 17.74 and 322 ± 18.78, respectively) during Day 0. Circulating E2 and TAC levels were significantly (P<0.05) higher in the LC-treated ewes (31.45 ± 1.53 pg/ml and 1.80 ± 0.13 mM/L, respectively) compared to those in the control ewes (21.20 ± 1.30 pg/ml and 0.98 ± 0.09 mM/L, respectively) during Day 0. Moreover, LC improved the colored pixel area of CLs (2038.14 ± 102.94 versus 1098 ± 82.39) and uterus (256.38 ± 39.28 versus 121.75 ± 11.36) and circulating P4 (2.99 ± 0.26 ng/ml versus1.67 ± 0.15 ng/ml) on Day 8. Values of RI of MUA were significantly lower in the LC group compared to the control one on Day 0 and Day 8 (0.48 ± 0.03 versus 0.72 ± 0.03 and 0.58 ± 0.03 versus 0.78 ± 0.02, respectively). In conclusion, LC incorporation in the OvSynch protocol enhanced the morphometrical and hemodynamic parameters of the ovarian structures and the uterus concomitantly with improvements in the TAC, E2, and P4 concentrations in ewes under hot summer conditions.

## Introduction

Sheep are a very important livestock species because they convert low-quality roughage, and feedstuffs into beneficial products for human consumption such as meat, milk, fiber, and hides [1]. However, several caveats such as heat stress (HS) could potentially affect their reproductive performances. HS is the state in which an animal’s body processes are engaged to maintain its body thermal equilibrium after being exposed to an intolerably higher temperature [2]. Sheep are one of the most susceptible species of livestock that could be greatly suffered from exposure to HS, especially during the summer months in tropical and subtropical countries such as Egypt [3, 4]. Much literature indicates the negative impacts of thermal stress on the reproductive performance of sheep [5, 6]. Reduced behavioral estrus, decreased incidence of estrus and intensity of estrus symptoms, and increased incidence of early embryonic mortalities are the most common outcomes of thermal stress, especially in the period between Day − 7 to Day 0 (ovulation time) in ewes [6].

Environmental HS could directly influence the fertility status of ewes through its effects on the hypothalamic-pituitary-adrenal axis (HPA), while the indirect effects of HS may include its influences on air temperature, decreased feed intake and water availability, and carbon dioxide levels in the atmosphere [6, 7]. Furthermore, exposure of sheep bodies to hyperthermia causes behavioral, physiological, and metabolic reactions, affecting reproductive success. HS negatively influences the quality of oocytes and follicular development. Summer HS lowers the potential fertility of cattle by influencing oocyte quality, follicular activity, and blood plasma progesterone (P4) levels [7–10]. For more instances, the exposure of sheep to HS before or during behavioral oestrus could induce a marked increase in the incidence of cytoplasmic vacuoles, cytoplasmic globules, ruptured oolemma, and cracked zona pellucida [6, 11].

Estrous synchronization (ES) can effectively improve sheep’s reproductive performance, shorten the estrus interval, and increase the pregnancy and lambing rates [12–14]. In addition, ES could facilitate artificial insemination and reduce the time and effort required for heat detection, especially, in large herds or farms. More importantly, Estrous synchronization protocols could reduce the effects of seasonal factors on ewe reproduction by extending the breeding season, adjusting the delivery time, and shortening the lambing cycle, all of which would lower the financial cost of breeding, result in more lambs, and gaining more economic profits [15–18]. A comprehensive understanding of ovarian secretary function in relation to the control of follicular development, the luteal phase of the cycle, and ovulation is necessary for synchronization protocol success [19]. Several hormonal schemes are established for the synchronization of estrus in sheep. The OvSynch protocol is one of the important GnRH-based protocols for estrous synchronization in sheep, being based on the incorporation of gonadotropins and prostaglandin F2 alpha (PGF_2α_). The OvSynch regimen offers reasonable pregnancy rates (> 80%) in ewes from tropical herds [20]. Indeed, the outcomes of estrous synchronization protocols may vary between different seasons; being lower during the hot summer compared to the temperate seasons [21]. Therefore, different reproductive strategies that aim to enhance the efficiency of estrous synchronization protocols are required to counteract the adverse effects of summer HS, especially on ewes. Incorporating a strong antioxidant may be needed to enhance the efficiency of the OvSynch protocol for estrous synchronization in ewes, especially under hot climatic conditions.

L-carnitine (LC) is a natural substance that is synthesized from the necessary amino acids (lysine and methionine). It arises from dietary sources (75%), as well as endogenous biosynthesis (25%) [22]. L-carnitine (LC) is a vitamin-like and bioenergetic amino acid that has an important role in cell energy production via fatty acids (long-chain) transport through mitochondrial membranes and subsequent synthesis of adenosine triphosphate via the beta-oxidation pathway [23]. L-carnitine protects the cell against different kinds of oxidative stress through its antioxidative properties, free radicals capturing, and lipid peroxidation inhibition [24–26]. We hypothesize that incorporating LC as a single administration could enhance the efficiency of the OvSynch protocol in ewes during hot climatic conditions.

The development of Doppler ultrasonography has resulted in enormous uses of this technology in various perspectives of animal reproduction practices [27–30] because it enabled researchers and veterinarians to assess both organ morphology and functions based on vascular perfusion [28, 30]. Color Doppler ultrasonography has been implemented for early discrimination of pregnancy status in buffaloes [31–34], cows [35, 36], and goats [37] and to assess the superovulation response in sheep [38] based on an assessment of ovarian and uterine vascularity. However, its potential usefulness to assess the estrous synchronization protocols was not investigated yet in sheep. The current study investigated the efficacy of LC administration on the efficiency of the OvSynch protocol for estrous synchronization in ewes during hot summer months. Follicular, luteal, and uterine blood perfusions were assessed utilizing color Doppler ultrasonography. In addition, circulating estradiol (E2), progesterone (P4) levels, and total antioxidant capacity (TAC) were measured.

## Materials and methods

This study was conducted on Ossimi ewes (*Ovis aries*), in the summer of 2022 at the educational farm (30°0′47.0016″N and 31°12′ 31.8708″E), Faculty of Veterinary Medicine, Cairo University, Giza, Egypt. Ossimi ewes are one of Egyptian fat-tailed sheep, raised mainly for meat and wool productions. This breed has low reproductive potential during summer [39, 40]. All procedures in this study were performed after obtaining the required consent and following guidelines established by the Animal Care and Ethical Use Committee, Faculty of Veterinary Medicine, Cairo University, for the use of animals (Protocol: Vet CU20-09-2022-481). In this regard, animals were released after finishing the study and were not exposed to any methods/steps of euthanization procedures.

### Animals’ management and design of experiment

Sixteen ewes (*Ovis aries*), aged 2.69 ± 0.35 years old (mean ± SD), and weighing 58.63 ± 2.42 kg (mean ± SD) were used in this study. All ewes were managed under natural daylight conditions and fed a maintenance-balanced ration of pelleted concentrate (a mixed formula of concentrate pellets; 14% crude protein and 6.39 MJ/kg diet as an energy requirement) and hay. According to the formulation of the diet, LC was not available as a beneficial element in the diet (since it should be rumen protected because it is easily degradable by rumen enzymes) [41]. Clean water and salt licks blocks were accessed *ad libitum*. All ewes were in good general health conditions, and no symptoms of any reproductive abnormalities were found during a thorough gynecological examination before or during the study. Ewes were regularly subjected to prophylactic measures including deworming and vaccinations against endemic diseases such as sheep pox, enterotoxaemia, and foot and mouth disease.

**Fig. 1:**
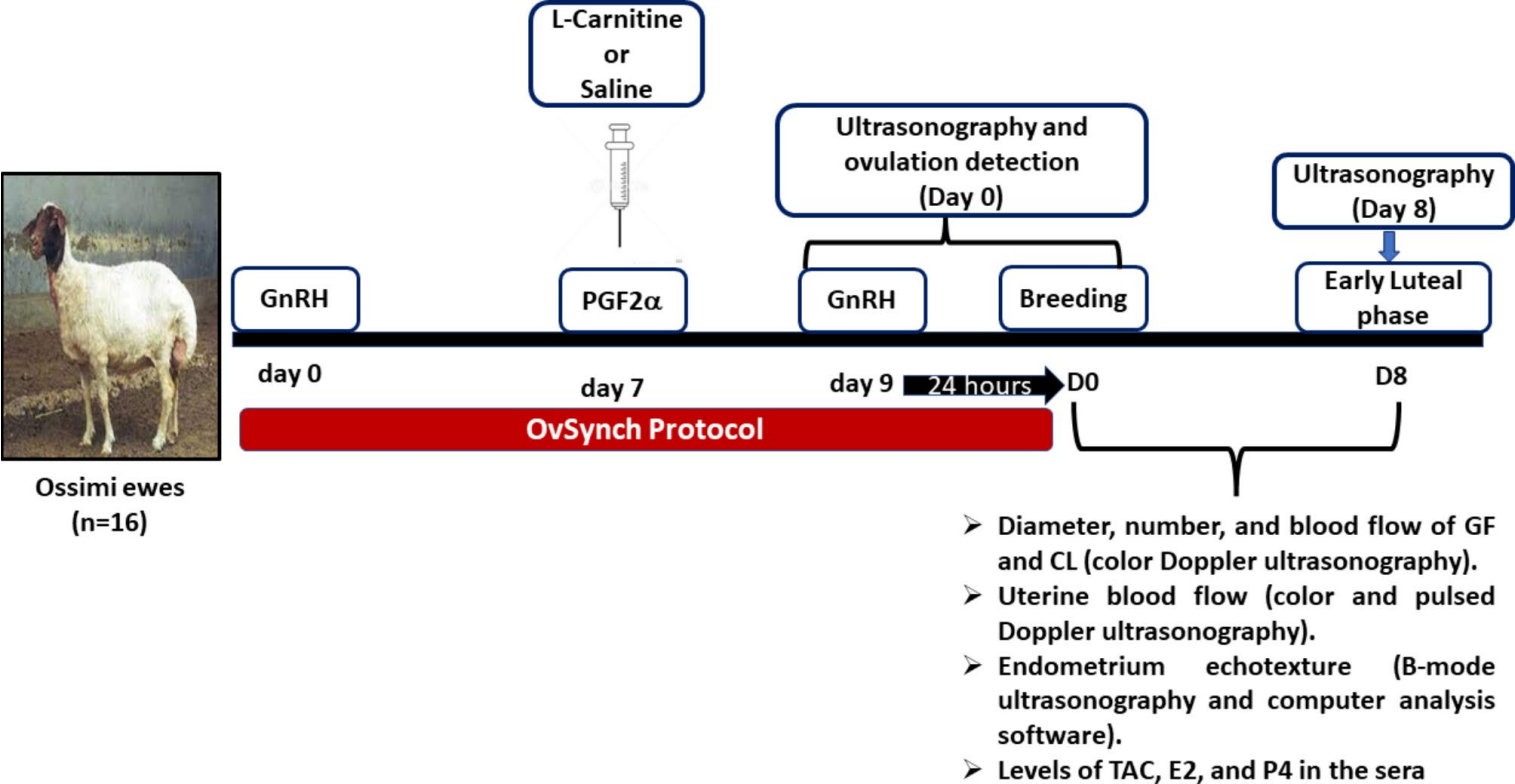
Schematic diagram of the experimental design of this study

The experimental design is illustrated in Fig. 1. Ewes were synchronized for estrus using the OvSynch protocol that includes the administration of two doses of 0.004 mg buserelin/head intramuscularly (Receptal; 0.004 mg buserelin/ml; Intervet International GmbH, Germany, MSD Animal Health) nine days apart. An intramuscular dose of prostaglandin F2 alpha (PGF_2α_) analog (250 µg of cloprostenol/head; Estrumate; 250 µg cloprostenol /ml; Vet Pharma Friesoythe Gmbh, Germany) was administered two days before the second shot of buserelin. A dose of LC (20 mg/kg body weight; L-Carnitine; 1000 mg/ ampoule; Mepaco Corporation, Egypt) [23, 42] was intravenously (IV) administered on the same day of PGF_2α_ administration to one group (n = 8; LC group), while another group (n = 8; control group) received the comparable volume of saline IV. Following the second dose of buserelin, ultrasonography was used to assess the number, size, and blood flow of the Graafian follicles (GF) during the estrous phase (Day 0). Two fertile rams were used for semen collection and insemination (500 × 10^6^ spermatozoa) of ewes in both groups as previously reported [43]. In brief, each ewe was restrained (with lifted hind limbs) and the insemination with fresh diluted semen was performed intracervical using a pipette attached to a syringe and the aid of a lubricated speculum.

Another ultrasonographic scanning was performed to assess the number, size, and blood flow of corpora lutea (CLs) on Day 8 post-ovulation (luteal phase) in both groups. Day 8 was selected to assess the CL’s blood flow, biometry, and secretary potency because it was considered the critical period for CL functionality (from Day 5 to Day 10) and development [44]. Then, a real-time B-mode ultrasonographic scanning was performed 35 days after ewes’ insemination to confirm pregnancy based on the appearance of the fetus, fetal fluids, and placentomes [37].

### Ultrasonographic assessment of the uterus and the ovarian response

All ultrasonographic scanning was performed by the same operator who has experience in reproductive ultrasonography in farm animals and by using a portable Doppler ultrasound device (ExaGO Doppler ultrasound, IMV, France). This device was supplemented with color Doppler mode and a linear transducer (5-7.5 MHz). All settings of the ultrasound scanner were standardized and fixed for all scanning procedures, the high pass filter was adjusted at 150 Hz, and the gate of the Doppler angle with the examined vessels was adjusted constantly at 1.5 mm with an insonation angle of less than 60°. Pulse repetition frequency (PRF) was 4000 Hz.

Each ewe was scanned by transrectal ultrasonography in a standing position following the previously established procedures [43, 45]. The linear rectal transducer had been modified by taping a wooden rod to the probe to control the manipulation of the transducer inside the rectum. The rectum was cleared of feces when necessary, and an ultrasonographic carboxymethyl cellulose gel (Echogel, IBE Technologies, Egypt) was applied to the transducer to facilitate its introduction into the rectum and improve the ultrasonographic imaging. After introducing the lubricated transducer into the rectum, it was slowly rotated 90° clockwise and 180° anticlockwise to image the entire reproductive tract by B-mode ultrasonography. In some cases, visualization of the uterus was enhanced by lifting the ventral body wall in front of the udder while conducting the scanning [43]. In this regard, the detection of the day of ovulation (Day 0) in each ewe was assessed by the disappearance of large antral follicles that were visualized at the previous transrectal ultrasonographic examination and later confirmed by the detection of CLs [45–47].

**Fig. 2:**
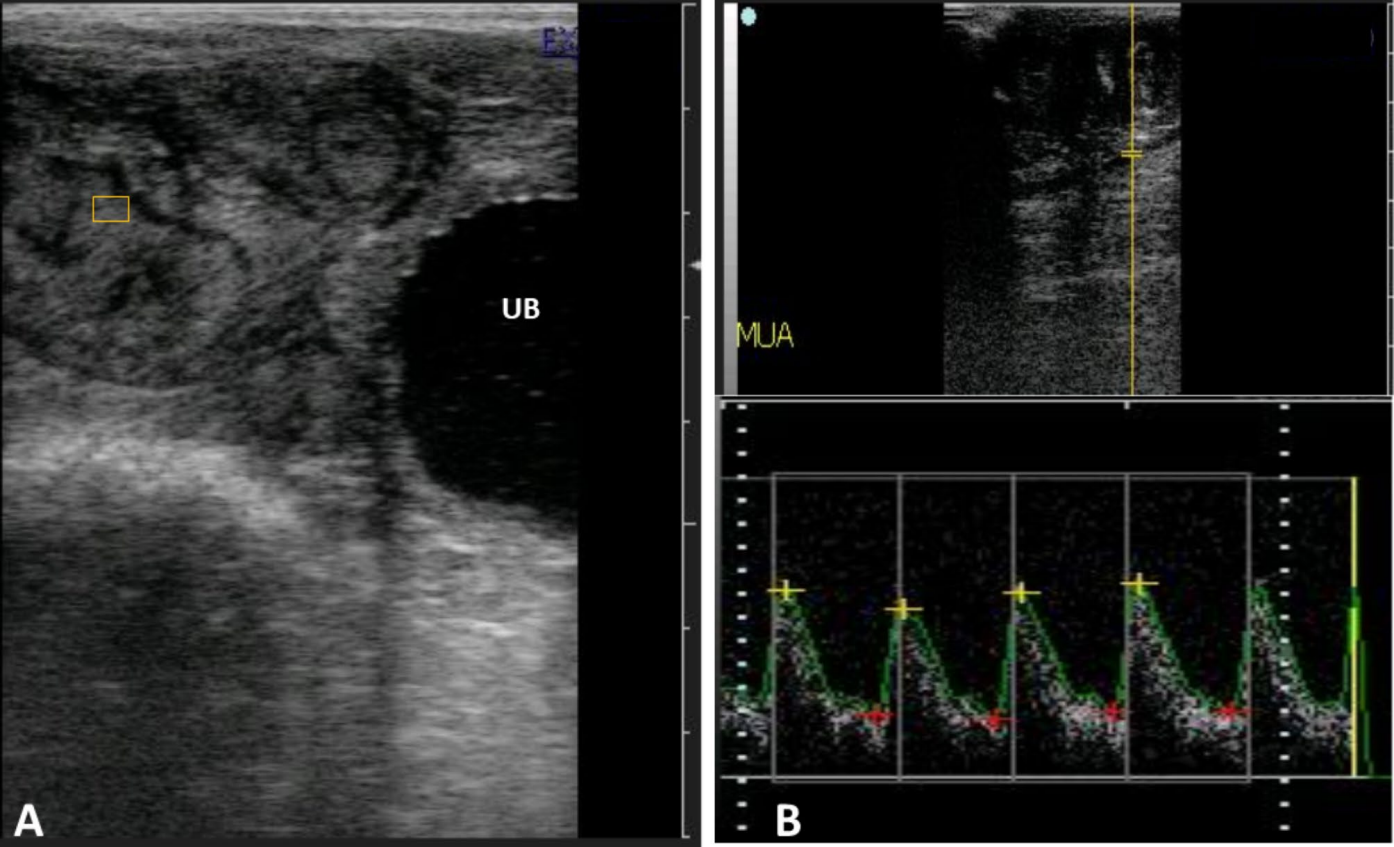
B-mode ultrasonographic appearance of the uterus of ewes for echotextural assessment of the endometrium (**A**) using a computerized image analysis program and pulsed Doppler assessment of blood flow within the uterine artery (**B**)

The number of ovarian structures (follicles and corpus luteum) and follicular and luteal blood flow (FBF and LBF, respectively) were examined on Day 0 and Day 8, respectively. In this regard, the ovary was visualized rectally by B-mode ultrasonography, and the diameters of the GF and CLs (mm) were determined using the electronic calipers. After visualization of ovarian structures by B-mode ultrasonography, the color Doppler mode was activated in slow continuous motion, constant color gain, velocity, and color-flow filter settings to display signals for blood flow in the observed GFs and CLs at Day 0 and Day 8, respectively. Similarly, the uterus was imaged by B-mode ultrasonography (Fig. 2A), and the good images on Day 0 and Day 8 were stored for further assessment of the echotexture of the uterus endometrium. Then, the color Doppler mode was activated to display signals for blood flow in the observed uterus. For assessment of the blood flow within the middle uterine artery (MUA), the uterus was imaged by B-mode ultrasonography, and the MUA was first recognized by B-mode ultrasonography as previously mentioned [48]. In brief, the uterine artery was located cranio-lateral to the bladder through a transrectal approach using the external iliac artery as a reference guide. The uterine artery was visualized in both directions in a cross or longitudinal section by positioning the transducer laterally and dorsally to the iliac artery branch.

Then, the color-pulsed Doppler approach was used to assess uterine blood flow by measuring the Doppler indices [resistive index (RI), pulsatility index (PI); and the systolic/diastolic ratio (S/D ratio)] of the blood flow within the middle uterine artery (Fig. 2B) as previously described [48]. Three minimum measurements were taken for each Doppler parameter for evaluating the blood flow within the MUA, and the mean values were considered. The ultrasound scanner automatically recorded and preserved the ultrasound images and all pertinent measurements.

**Fig. 3:**
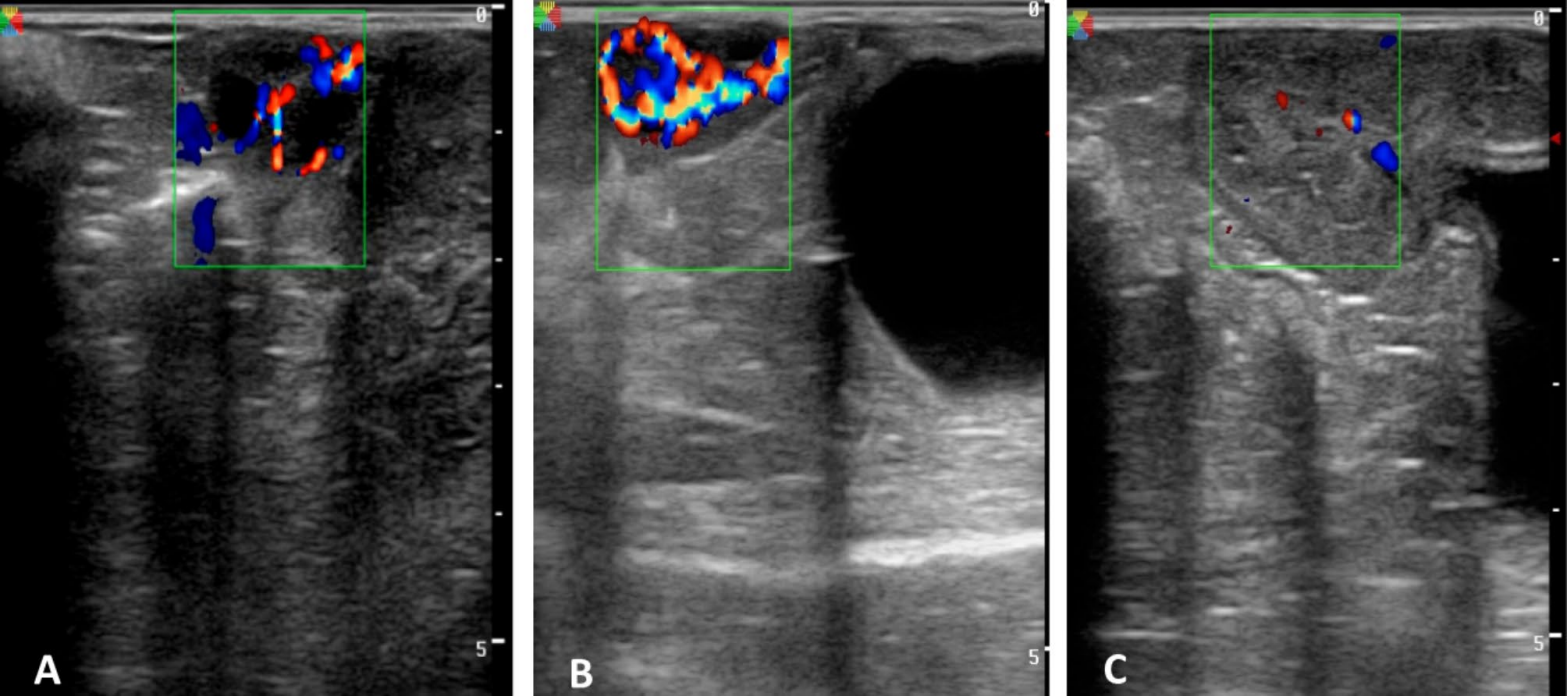
Color Doppler imaging of follicular blood flow (**A**), luteal blood flow (**B**), and uterine blood flow (**C**) to assess the blood flow perfusions area (pixels), integrated density of the colored areas, and the perimeter of the colored areas using a computerized image analysis program in ewes

The good color-mode images of the GFs, CLs, and uterus were selected for computed image analysis using software (Adobe PhotoShop CC software (1990–2013, Adobe Systems). Colored areas of the blood flow of the GFs (FBF), corpus luteum (LBF), and uterus (UBF) were counted in pixels to assess the blood flow perfusion area (s) in pixels using an image analysis program (Adobe Photoshop CC software, Adobe Systems, USA) as described previously [33, 34]. In brief, good images of color Doppler of the GFs (Fig. 3A), CLs (Fig. 3B), and uterus (Fig. 3C) were analyzed for the blood flow perfusions area (s) in pixels, integrated density (IGD) of the colored areas, and the perimeter of the colored areas using an image analysis program. At least three images of color Doppler of each tissue were analyzed, and the mean values were considered.

### Echotexture analysis of the uterus by the computed-image analysis software

Good images of B-mode ultrasonography of the uterus (at Day 0 and Day 8) were selected for further assessment of the echotexture of the uterus endometrium (pixel intensity and echotextural heterogeneity) using an image analysis software program (Adobe Photoshop CC software, Adobe Systems, USA) following the previously described reports [33, 49]. In brief, the ultrasonographic images of the uterus were assessed using the spot technique. A total of 4 square-shaped spots (5 mm × 5 mm) were placed on each saved image of the endometrium (Fig. 2A), except for artifacts. The pixel intensity of the endometrium represents the average pixel values within the selected area of the tissue based on a reverse scale of gray shades (one–255); so that number one is pointed to as black and 255 is referred to as white [50, 51]. In this regard, at least three B-mode images of the uterus were analyzed, and the mean values were considered.

### Blood sampling and hormonal analysis

On the same days of ultrasonographic assessment, blood samples (five ml) were drawn (via the jugular vein into evacuated tubes from each ewe). Then, blood samples were kept at room temperature for 20 min, and the sera were harvested by centrifugation of blood samples at 3200 rpm for 15 min. The serum samples were then separated, aliquoted, and kept at -20 °C for measuring concentrations of E2 (pg/ml), P4 (ng/ml), and TAC (mM/L). Concentrations of P4 (EIA-1561) and E2 (EIA-2693) were analyzed using the commercial kits as described by the manufacturers (BioCheck, Inc. Foster City, CA, USA) [27, 52]. The intra-assay coefficients for measuring P4 and E2 were 6.80% and 5.5%, respectively, while the sensitivities were 0.05 ng/ml and 9.72 pg/ml, respectively. Levels of TAC (mM/L) were measured photometrically using a commercial kit (Bio-diagnostics, Giza, Egypt) via a spectrophotometer adjusted at a wavelength of 505 nm and following the manufacturer’s instruction with an intra-assay coefficient of 3.4% and at 0.04 mM/L as a least detectable concentration of TAC [27]. The measurement of TAC depends on the ability of the sample’s antioxidant content to suppress an exogenously supplemented hydrogen peroxide (with a defined amount), while the ability to convert 3,5, dichloro2- hydroxy benzenesulfonate to a colored product determines residual hydrogen peroxide.

### Statistical analysis

Data were presented as mean ± standard error of the mean (SEM). Firstly, the data were checked for normality using the Shapiro–Wilk test [53]. The differences between LC and control groups in the studied parameters at either Day 0 or Day 8 were analyzed using the student t-test. All the statistical procedures were performed utilizing GraphPad Prism5 software for all the studied parameters. Correlations between various parameters were studied using the correlations coefficient. A probability value of less than 0.05 was considered significant, while the values between 0.1 and 0.05 were considered tended.

## Results

On Day 0 (Table 1), significant increases in the total number of follicles and the average number of dominant follicles >5 mm/ewe were found in the LC group (5.75 ± 0.60 and 3.25 ± 0.45, respectively) compared to those in the control group (2.25 ± 0.21 and 1.75 ± 0.18, respectively). In addition, there were significant increases in the colored pixel area (392.84 ± 31.86), and the integrated density (27665.40 ± 2415.87) of the GF blood flow in the LC group compared to its values in the control group (226.25 ± 17.74 and 14603.25 ± 1302.66, respectively) during the estrous phase. No significant differences were found in the average size of the GFs and the perimeter of the colored pixel area of the FBF between both groups. Regarding uterine echotexture and hemodynamics, results revealed significant increases in the colored pixel area, IGD, and the perimeter of uterine colored areas during the estrous phase in the LC group (712.50 ± 46.88, 63,439 ± 2540, and 183.00 ± 11.25, respectively) compared to that in the control one (322 ± 18.78, 18,759 ± 2355, and 109.80 ± 8.66, respectively). Similarly, Doppler indices (RI, PI, and S/D) of the blood flow within the MUA showed significant decreases in the LC group (0.48 ± 0.03, 1.32 ± 0.07, and 1.42 ± 0.05, respectively) compared to those in the control group (0.72 ± 0.03, 2.02 ± 0.15, and 2.93 ± 0.19, respectively) during the estrous phase. However, the pixel intensity of the endometrial echotexture and heterogeneity showed non-significant alterations between the LC and control groups.

**Table 1:** Morphometrical and hemodynamic parameters of the ovarian structures and uterus during the estrous phase (Day 0) in ewes that received the OvSynch protocol incorporated with L-Carnitine administration (LC group; n = 8) and ewes that received the OvSynch protocol without the incorporation of L-Carnitine administration (Control group; n = 8) during the summer months

Parameters	Control group (n = 8)	LC group (n = 8)	P-value
Total number of follicles/ewe	2.25 ± 0.21	5.75 ± 0.60	P < 0.05
Number of dominant follicles >5 mm	1.75 ± 0.18	3.25 ± 0.45	P < 0.05
Size of DF (mm)	5.96 ± 0.46	7.28 ± 0.52	P > 0.05
Pixel intensity of endometrial echotexture	66.49 ± 4.61	62.51 ± 2.64	P > 0.05
Echotexture heterogeneity of endometrial echotexture	21.38 ± 1.29	20.60 ± 1.64	P > 0.05
Colored pixel area of FBF	226.25 ± 17.74	392.84 ± 31.86	P < 0.05
Integrated density of colored area of FBF	14603.25 ± 1302.66	27665.40 ± 2415.87	P < 0.05
Perimeter of colored area of FBF	98.80 ± 7.14	122.69 ± 10.13	P > 0.05
Colored pixel area of UBF	322 ± 18.78	712.50 ± 46.88	P < 0.05
Integrated density of colored area of UBF	18,759 ± 2355	63,439 ± 2540	P < 0.05
Perimeter of colored area of UBF	109.80 ± 8.66	183.00 ± 11.25	P < 0.05
RI of blood flow within the MUA	0.72 ± 0.03	0.48 ± 0.03	P < 0.05
PI of blood flow within the MUA	2.02 ± 0.15	1.32 ± 0.07	P < 0.05
S/D of blood flow within the MUA	2.93 ± 0.19	1.42 ± 0.05	P < 0.05

Abbreviations: DF: dominant follicles, FBF: follicular blood flow, UBF: uterine blood flow, RI: resistive index, PI: pulsatility index, MUA: middle uterine artery; S/D: systolic/diastolic ratio

On Day 8 **(**luteal phase; Table 2), the number of the detected CLs tended (P = 0.08) to be higher in the LC group (1.80 ± 0.22) compared to that in the control group (1.2 ± 0.16). No significant differences (P > 0.05) were found in terms of the size of CL (mm), number of follicles, and size of DF >4 mm. Significant (P < 0.05) increases in the colored pixel area of CLs and the IGD of the colored area of CLs were noticed in the LC group (2038.14 ± 102.94 and 161370.38 ± 14739.49, respectively) compared to those in the control one (1098 ± 82.39 and 49029.88 ± 5482.76, respectively). Regarding uterine morphometry and hemodynamics, results revealed significant increases in the colored pixel areas, IGD of colored areas, and the perimeter of colored area of the endometrium in the ewes that received LC administration in the OvSynch protocol (256.38 ± 39.28, 18831.63 ± 2411.83, and 106 ± 10.21 mm, respectively) compared to that in the control ewes (121.75 ± 11.36, 12173.50 ± 1182.65, and 67.88 ± 5.96 mm, respectively). Similarly, the RI of the blood flow within the MUA had lesser values in the LC received ewes (0.58 ± 0.03) than the control ones (0.78 ± 0.03). Although the PI of blood flow within the MUA tended (P = 0.07) to be high in the control group (1.72 ± 0.12) compared to that in the LC group (1.19 ± 0.12), the S/D ratios were not significantly (P > 0.05) differed between the two groups. Regarding echogenicity of the endometrium, significant (P < 0.05) decreases in the pixel intensity of endometrial echotexture were found in the LC group (54.60 ± 1.71) compared to the control one (69.16 ± 3.18), while the echotexture heterogeneity values weren’t significantly differed.

**Table 2:** Morphometrical and hemodynamic parameters of the ovarian structures and uterus during the luteal phase (Day 8) in ewes that received the OvSynch protocol incorporated with L-Carnitine administration (LC group; n = 8) and ewes that received the OvSynch protocol without the incorporation of L-Carnitine administration (Control group; n = 8) during the summer months

Parameters	Control group (n = 8)	LC group (n = 8)	P-value
Number of CLs	1.2 ± 0.16	1.80 ± 0.22	P = 0.08
Size of CL (mm)	11.23 ± 0.66	10.68 ± 0.49	P > 0.05
Size of DF (> 4 mm)	3.63 ± 0.20	4.61 ± 0.25	P > 0.05
Number of follicles	2.25 ± 0.19	2.15 ± 0.15	P > 0.05
Pixel intensity of endometrial echotexture	69.16 ± 3.18	54.60 ± 1.71	P < 0.05
Echotexture heterogeneity of endometrial echotexture	22.72 ± 1.03	25.67 ± 2.19	P > 0.05
Colored pixel area of LBF	1098 ± 82.39	2038.14 ± 102.94	P < 0.05
Integrated density of colored area of LBF	49029.88 ± 5482.76	161370.38 ± 14739.49	P < 0.05
Perimeter of colored area of LBF	221.25 ± 30.16	280 ± 20.65	P > 0.05
Colored pixel area of UBF	121.75 ± 11.36	256.38 ± 39.28	P < 0.05
Integrated density of colored area of UBF	12173.50 ± 1182.65	18831.63 ± 2411.83	P < 0.05
Perimeter of colored area of UBF	67.88 ± 5.96	106 ± 10.21	P < 0.05
RI of blood flow within the MUA	0.78 ± 0.03	0.58 ± 0.03	P < 0.05
PI of blood flow within the MUA	1.72 ± 0.12	1.19 ± 0.12	P = 0.07
S/D of blood flow within the MUA	4.02 ± 0.21	3.01 ± 0.26	P > 0.05

Abbreviations: DF: dominant follicles, LBF: luteal blood flow, UBF: uterine blood flow, RI: resistive index, PI: pulsatility index, MUA: middle uterine artery; S/D: systolic/diastolic ratio

Biochemically (Table 3), TAC (mM/L) and E2 (pg/ml) levels increased significantly (P < 0.05) during Day 0 in the LC group (1.80 ± 0.13 mM/L and 31.45 ± 1.53 pg/ml, respectively) compared to that in the control one (0.98 ± 0.09 mM/L and 21.20 ± 1.30 pg/ml, respectively), while P4 levels did not significantly (P > 0.05) differ at this stage. During Day 8, the levels of P4 (ng/ml) were significantly higher in the LC-received ewes (2.99 ± 0.26 ng/ml) compared to those in the control ewes (1.67 ± 0.15 ng/ml), while the TAC (mM/L) and E2 levels did not significantly (P > 0.05) differ at this stage.

**Table 3:** Levels of total antioxidant capacity (TAC; Mm/l), estradiol (pg/ml), and progesterone (ng/ml) in ewes that received the OvSynch protocol incorporated with L-Carnitine administration (LC group; n = 8) and ewes that received the OvSynch protocol without the incorporation of L-Carnitine administration (Control group; n = 8) during the summer months

Parameters	Control group (n = 8)	LC group (n = 8)	P-value
TAC (mM/L) levels during Day 0	0.98 ± 0.09	1.80 ± 0.13	P < 0.05
TAC (mM/L) levels during Day 8	2.15 ± 0.14	2.74 ± 0.17	P > 0.05
Estradiol (pg/ml) levels during Day 0	21.20 ± 1.30	31.45 ± 1.53	P < 0.05
Estradiol (pg/ml) levels during Day 8	7.91 ± 0.53	9.20 ± 0.49	P > 0.05
Progesterone (ng/ml) levels during Day 0	0.41 ± 0.02	0.42 ± 0.03	P > 0.05
Progesterone (ng/ml) levels during Day 8	1.67 ± 0.15	2.99 ± 0.26	P < 0.05

In this regard, high correlations (P< 0.05) were found between the total number of follicles/ewe, the number of dominant follicles, the colored pixel area of FBF, integrated density of the colored area of FBF, and blood flow parameters of the uterus (colored pixel area of UBF, integrated density of colored area of UBF, perimeter of colored area of UBF, and Doppler indices of blood flow within the MUA) with E2 levels. Also, high correlations (P< 0.05) were found between the colored pixel area of LBF, integrated density of the colored area of LBF, pixel intensity of endometrial echotexture, and some of the blood flow parameters of the uterus (colored pixel area of UBF, integrated density of colored area of UBF, perimeter of colored area of UBF, and RI of blood flow within the MUA) with P4 levels (Table 4). In this study, ultrasonographic examination revealed that 6/8 ewes were pregnant in the LC-treated group compared to 4/8 ewes in the control group.

**Table 4:** Results of correlation coefficients between the ultrasonographic and biochemical parameters during the estrous phase (Day 0) and early luteal phase (Day 8) in Ossimi ewes of both groups during the summer months (n = 16)

Parameters on Day 0	E2	P4	TAC
Total number of follicles/ewe	0.857^**^	0.035	0.765^*^
Number of dominant follicles >5 mm	0.685^**^	0.154	0.664^*^
Size of DF (mm)	0.558^*^	0.095	0.388
Pixel intensity of endometrial echotexture	-0.352	0.186	-0.258^*^
Echotexture heterogeneity of endometrial echotexture	0.335	0.225	0.354
Colored pixel area of FBF	0.895^**^	0.098	0.667^*^
Integrated density of colored area of FBF	0.785^**^	0.176	0.699^*^
Perimeter of colored area of FBF	0.863	0.324	0.710^*^
Colored pixel area of UBF	0.776^**^	0.307	0.678^**^
Integrated density of colored area of UBF	0.688^**^	0.245	0.742^*^
Perimeter of colored area of UBF	0.695^**^	0.178	0.635^*^
RI of blood flow within the MUA	-0.568^**^	0.248	-0.854^**^
PI of blood flow within the MUA	-0.665^**^	0.175	-0.841^**^
S/D of blood flow within the MUA	-0.606^**^	0.167	-0.799^*^
**Parameters on Day 8**	**E2**	**P4**	**TAC**
Number of CLs	0.218	0.556^*^	0.288
Size of CL (mm)	0.125	0.453	0.290
Size of DF (> 4 mm)	0.456	-0.314	0.357
Number of follicles	0.456	0.386	0.421
Pixel intensity of endometrial echotexture	-0.256	-0.673^*^	-0.336
Echotexture heterogeneity of endometrial echotexture	0.208	-0.219	0.183
Colored pixel area of LBF	0.086	0.777^**^	0.433
Integrated density of colored area of LBF	0.117	0.793^**^	0.389
Perimeter of colored area of LBF	0.097	0.414^*^	0.366
Colored pixel area of UBF	0.335	0.563^*^	0.313^*^
Integrated density of colored area of UBF	0.479	0.486^*^	0.331
Perimeter of colored area of UBF	0.278	0.438^*^	0.388
RI of blood flow within the MUA	-0.217	-0.576^*^	-0.424
PI of blood flow within the MUA	-0.193	-0.588^*^	-0.386
S/D of blood flow within the MUA	-0.187	-0.414	-0.277

^*^P < 0.05 for the correlations and ^**^P < 0.01 for the correlations

## Discussion

Climatic changes have negatively impacted the productive and reproductive performances of livestock all over the globe, especially in tropical and subtropical countries such as Egypt. This study is very important because it discussed, for the first time, the influence of LC incorporation in the OvSynch protocol of ewes under hot climatic conditions of the summer season. The results revealed the stimulatory effect of LC administration on the morphometrical and hemodynamic parameters of the ovarian structures and the uterus at Day 0 and Day 8. In the present study, Doppler indices of UBF were significantly lower in the LC group compared to the control group. Doppler indices are negatively correlated with vascular perfusion of tissue downstream [50]. Decreased Doppler indices are considered to have resulted from increased blood perfusion and refer to the continuous oxygen and nutrients supplied to the respective organ [54–56]. In addition, the LC incorporation increased the TAC and E2 levels on Day 0 and P4 levels on Day 8. These effects might be attributable to one of three possible explanations. First, LC may have stimulatory effects on cardiovascular functions [57–59], reflected in the enhancement of follicular, luteal, and uterine hemodynamics. It was noted that LC could facilitate the transport of long-chain fatty acids into the mitochondrial matrix, triggering cardioprotective effects through reduced oxidative stress, inflammation, and necrosis of cardiac myocytes [59]. Moreover, LC can regulate calcium influx, the integrity of the endothelium, the release of intracellular enzymes, and membrane phospholipid content for prolonged cellular homeostasis [59]. LC also has direct vasodilating and positive inotropic effects on the cardiovascular system. In dogs, LC administration increased coronary blood flow by about 60% and reduced coronary vascular resistance by 25% [57].

The second explanation may be attributable to the strong antioxidant property of LC, as reported in previous literature [41, 60–63]. In this study, the levels of TAC increased significantly at Day 0 in the LC-treated ewes compared to the control ewes. Similarly, the study by Canbolat [62] reported the stimulatory effects of LC on various oxidative stress biomarkers, including malondialdehyde, total antioxidant status, and total oxidative stress in various organs (kidney, liver, and heart) in the serum of rats. Moreover, LC supplementation during in vitro maturation of sheep oocytes reduced oxidative stress-induced embryotoxicity by decreasing intracellular reactive oxygen species and increasing intracellular glutathione, which in turn improved the developmental potential of oocytes and embryos [63]. Dietary supplementation of rumen-protected LC during the transition period could improve the productivity of high-producing dairy cows during the early post-partum period through modulating metabolic indicators as indexed in energy metabolism and liver functionality [41].

The third explanation might be attributable to the direct effect of LC on the hypothalamic-hypophyseal gonadal axis [64, 65]. Since it is abundant in the hypothalamus, LC could affect neuronal activity and thus the hypothalamic-pituitary-gonadal (HPG) axis to exert its impact on female reproduction [65]. LC improved folliculogenesis and the number of antral follicles in a mouse model [66]. In the current study, the LC-treated group showed a high number of 5 mm in diameter follicles and induced high levels of E2 at Day 0. In women, LC supplementation along with clomiphene citrate treatment improved both ovulation (64.4% vs.17.4%; p < 0.0001) and pregnancy rates (51.5% vs. 5.8%; p < 0.0001) in clomiphene-resistant women with polycystic ovaries [67]. Also, LC supplementation improved the number and rate at which the stimulated follicles developed (to a diameter of ≥ 17 mm for induction of ovulation), increased beta-oxidation and oocyte maturation, and increased serum levels of both E2 and P4 [67]. In mice, LC treatment increased the number of mature oocytes and reduced the percentage of degraded oocytes [68]. Samland et al. [69] examined the effect of dietary supplementation with LC on the ovulation and fertilization rates of gilts. After two weeks, the gilts fed with added LC attained a high ovulation rate.

The pregnancy rate was higher in the LC-treated ewes compared to the control ewes. LC has been reported to maintain the cellular energy of oocytes [70], reduce oxidative stress conditions [71], and minimize cell apoptosis and DNA damage, which are necessary for the proper growth of oocytes and the in vitro developmental competence of blastocysts in mice [72]. As discussed in previous literature, the cumulus-oocyte complex and lipid metabolism are prime regulators of oocyte maturation [73]. HS could impair lipid transport and disrupt the energetic metabolism of the oocyte and granulosa cells [74, 75]. In vitro, acute HS (43 °C) triggered oxidative stress-mediated apoptosis in cultured bovine granulosa cells and impaired oocyte developmental competence [76]. LC helps in the metabolism of lipids inside the cumulus-oocyte complex by transferring fatty acids into the mitochondria and facilitating the beta-oxidation pathway [77]. LC could directly affect the GFs by increasing oocyte energy production and effectively quench free radicals to protect them against oxidative damage. Moreover, LC could indirectly affect the GFs through its effect on the HPG axis and its secretory hormones [78].

The advent of image software grew the diagnostic potentials of ultrasonography and reduced the variability between observers of an ultrasound image through the assessment of changes in the organ’s echotexture [51, 79]. Monitoring changes in uterine echotexture using computer-image analysis of pixel intensity and integrity is thought to be an excellent predictor of histomorphology and chemical composition of the endometrium in livestock [49, 80, 81]. Some studies demonstrated the usefulness of evaluation of corpus luteum and uterine echotexture/ultrasonogram for determining pregnancy status [82] and postpartum uterine involution and its relationship to various infertility problems in various animal species such as in cows [49], sows [83], ewes [80, 81], and goats [84]. Significant decreases in the pixel intensity of the endometrium echotexture that were found in the LC group during Day 0 could be attributable to the increased UBF that could enhance the vascular permeability and increases in the endometrial fluid content, which in turn, leads to decreases in the pixel intensity of the endometrial echotexture. On the other hand, the increased levels of E2 that were noted during Day 0 in the LC-treated ewes might be in part related to the increased number of DF >5 mm that could participate in the E2 production [85]. Rather than the direct influence of LC on ovarian and uterine hemodynamics, the increased FBF and UBF noted in the LC group might be attributable to the effect of increased levels of E2, which has a vasodilatory role [86]. In women, the exogenous administration of E2 increased the blood flow of the uterus and ovary [87]. The vasodilatory effect of E2 may be mediated through intracellular signaling that involves decreases in the calcium uptake of potential sensory channels by the E2 receptors in the tunica media and E2 in the uterine arteries [88].

Importantly, increased levels of P4 found in the LC-treated ewes at Day 8 may be related to the increased LBF that could positively influence the secretory function of CLs as previously reported in buffaloes [34, 89], cows [36, 90], goats [37, 47], and sheep [46]. The current study revealed strong correlations between the LBF parameters and P4 concentration during Day 8. Previous reports also indicated the close associations between the increased LBF and its greater potency for P4 secretion, and in turn, the greater likelihood of pregnancy establishment and continuous embryonic development [34, 91–93].

There were some trials to improve the efficiency of the OvSynch regimen for estrous synchronization in dairy cattle, such as a presynchronization with a GnRH dose six days before initiation of the OvSynch or an addition of a second PGF_2α_ treatment [94]. However, the issues of low embryo implantation rate and significant pregnancy losses remain unresolved [95] despite advancements in the aforementioned methods in dairy cattle. This study is the first that demonstrates the beneficial effects of LC incorporation in the OvSynch protocol on morphometrical and hemodynamic parameters of the ovarian structures and the uterus in ewes under tropical summer conditions. Concomitantly, there were improvements in the circulating TAC, E2, and P4. The repeatability of LC incorporation in oestrous synchronisation protocols in sheep could be acceptable since the interestrous interval is about 17 days. However, further research should be performed to assess the outcomes. Overall, these findings may indirectly point out new insights into the potential improvement of the efficacy of the OvSynch regimen on the ewe’s fertility. However, performing this modification on many ewes might be needed in a further study to assess the fertility outcomes (represented by the pregnancy rate and kidding rates).

## Conclusion

Incorporating LC in the OvSynch protocol substantially enhanced the follicular blood flow, uterine blood flow, and circulating E2 and TAC during the estrous phase (Day 0). Moreover, it improved the luteal blood flow, uterine blood flow, and circulating P4 on Day 8 of the luteal phase.

## Data Availability

The datasets used and/or analysed during the current study are available from the corresponding author upon reasonable request.
